# The importance of Vitamin-D and Neutrophil-Lymphocyte Ratio for Alzheimer’s Disease

**DOI:** 10.12669/pjms.39.3.7024

**Published:** 2023

**Authors:** Ahmet Evlice, Zeynep Selcan Sanli, Pinar Bengi Boz

**Affiliations:** 1 Ahmet Evlice, MD. Department of Neurology, Faculty of Medicine, Cukurova University, Adana, Turkey; 2 Zeynep Selcan Sanli, MD., Faculty of Medicine, Neurology Clinic Health Sciences University Adana, Adana City Training and Research Hospital, Adana, Turkey; 3 Pinar Bengi Boz, MD., Faculty of Medicine, Neurology Clinic Health Sciences University Adana, Adana City Training and Research Hospital, Adana, Turkey

**Keywords:** Alzheimer’s disease, Ischemia, Vitamin D, Neutrophil–lymphocyte ratio

## Abstract

**Objective:**

Ischemia and inflammation play a role in the pathophysiology of Alzheimer’s disease (AD). Plasma neutrophil–lymphocyte ratio (NLR), and 25- hydroxyvitamin D (vitamin D) were used as a biomarker for inflammation and atherosclerosis. The present study aimed to investigate a link between NLR, vitamin D and ischemia in AD.

**Methods:**

The subjects with AD and control groups were enrolled to this retrospective study between 2017-2022 at Cukurova University Hospital. The cognitive assessment (MMSE), and blood tests (NLR, vitamin D) were collected from all subjects. In first part of the study, AD (n:132) and the control group (n:38) were compared. In second part of the study, magnetic resonance imaging (MRI) was used for evaluating ischemic lesions with scoring method of Fazekas. The control group (n:38) and AD subjects with mild ischemic lesions (Fazekas-1 and Fazekas-2) (n:64) were excluded. AD subjects with severe ischemic lesions (Fazekas-3) (n:34) and without ischemic lesions (Fazekas-0) (n:34) were compared again. SPSS 20.0 was used for all analyses. The threshold for statistical significance was set at 0.05.

**Results:**

In the first part of the study, 132 AD patients (69 female and 63 male; mean age 70.83±9.35 (49-87) and age-matched 38 controls were compared. The mean NLR in AD [2.96±2.46 (1.17-19.43)] was higher than the control group [1.9±0.66 (0.9-3.56)] (p=0.005). In the second part of the study, the mean Vitamin D of Fazekas-3 AD group [16.15±9.64 (4.7-35)] was lower than Fazekas-0 AD group [16.27±6.81(4.6-29.7)] (p=0.024).

**Conclusion:**

NLR was higher in AD while there was no difference between the Fazekas-0 and Fazekas-3 AD groups. Vitamin D was lower in the Fazekas-3 AD group. These data suggested that NLR increased independently of ischemia in AD. Also vitamin D deficiency could trigger ischemia in AD.

## INTRODUCTION

Dementia is a general term that illustrates cognitive decline in brain function.^1^ Alzheimer’s disease (AD), the most common cause of dementia, is a progressive neurodegenerative disorder with uncertain pathogenesis. AD has different biomarkers^2^ (amyloid/tau in cerebrospinal fluid, amyloid/tau positron emission tomography), but they are not available in all centers, so many studies perfromed for detecting accessible plasma biomarkers. For example, plasma neutrophil–lymphocyte ratio (NLR) was found higher^3^ and, plasma 25- hydroxyvitamin D (vitamin D) was lower^4^ in AD. Previous studies have highlighted that NLR and hypovitaminosis D can be an indicator of systemic inflammation.^3^-^5^ Chronic inflammation in the arterial wall plays a crucial role in progression of atherosclerosis.^6^ NLR can be higher in patients with atherosclerosis^7^, and hypovitaminosis D has a direct link with the prevalence of vascular mortality.^8^ As a result, NLR and Vitamin D have links to both inflammation and atherosclerosis. According to the vascular hypothesis of AD, vascular risk factors can result in dysregulation of the neurovascular unit and hypoxia. Hypoxia may increase neuronal dysfunction and neurodegeneration, resulting in AD.^9^ Neuroimaging studies suggest that white matter hyperintensities explained by vascular mechanisms occur frequently in AD.^10^ The presence of white matter lesions (WML) in the brain is one of the signs of ischemia. Until now there have been a few controversial studies about relationship between NLR^11^,^12^, vitamin D^13^,^14^, and WML. The present study aimed to investigate a link between NLR, vitamin D, and WML in Alzheimer’s disease.

## METHODS

The study was designed as a case and control group. The cases with AD (n:132) and the control group (n:38) were enrolled to this retrospective study in the Department of Neurology at Cukurova University Hospital, Turkey between 2017-2022. AD was diagnosed according to the National Institute of Neurological and Communicative Disorders and Stroke and the Alzheimer’s Disease and Related Disorders Association (NINCDS-ADRDA) criteria^2^ and Clinical Dementia Rating Scale (CDR).^15^ CDR is a severity rating range along a 5-point scale. CDR-0: no cognitive impairment CDR-0.5: mild cognitive impairment CDR-1: mild dementia CDR-2: moderate dementia CDR-3: severe dementia. The AD group contained subjects with cognitive impairment who has mini-mental state examination (MMSE)^16^ score ≤ 24 and CDR≥1. The control group contained elderly subjects with amnesia but without cognitive impairment (MMSE score > 24 and CDR=0).

The data of cognitive assessment (MMSE), routine blood tests (complete blood count, NLR, vitamin D, vitamin B12, folate) and magnetic resonance imaging (MRI) of subjects were collected.

### MRI:

1.5 T MRI scanner (Magnetom Avanto, Siemens Medical Solutions, Erlangen, Germany) was used to image the brain. T2-weighted FLAIR images were utilized to define WML using the semi-quantitative visual rating scale developed by Fazekas et al.^17^ WML was rated with Fazekas scale from 0 to 3. Fazekas-0: normal, Fazekas-1: punctuating foci, Fazekas-2: starting confluence of foci, and Fazekas-3: large confluent regions.

### NLR:

Complete blood count analysis was performed with Beckman Coulter Gen-S (High Wycombe, UK) automated analyzer. It was divided by the neutrophil count by the lymphocyte count to get NLR.

### Vitamin-D:

Using a radioimmunoassay, the concentration in the blood was calculated in nanomoles (division by 2.496 to convert to ng/ml) (DiaSorin Inc., Stillwater, MN, USA).

### Exclusion Criteria:

- History of stroke,
- Dialysis, Coronary heart disease,
- Severe liver failure, Malignant disease,
- Active infections, active inflammatory disorders,
- Participants who cannot be communicated due to hearing loss or aphasia.

The study protocol was approved by the Non-Interventional Clinical Research Ethics Committee of Cukurova University (Protocol no: 2022/123-11). The standard protocol at Neurology Department contains acquiring signed informed consent from all patients for evaluation of their medical records for any reason.

After the data of AD (n:132) and control group (n:38) were compared, AD subjects with mild ischemic lesions (Fazekas-1 and Fazekas-2) (n:64) and the control group were excluded.

AD subjects with severe ischemic lesions (Fazekas-3) (n:34) and without ischemic lesions (Fazekas-0) (n:34) were compared again.

### Statistical analysis:

Numeric values used to express categorical variables while the mean and standard deviation were used to describe continuous variables. To compare categorical variables between the groups, the Chi-square test was performed. The Shapiro Wilk test was used to verify the normality of the distribution for continuous data. Mann-Whitney U-test was used to compare continuous variables between two groups. To evaluate the correlations between measurements Spearman Rank Correlation Coefficient was used. The best cutoff thresholds for NLR and vitamin D to predict, respectively, Alzheimer’s disease/Control and Fazekas zero/Fazekas three, were determined by a receiver operator characteristic (ROC) curve analysis. The statistical program IBM SPSS Statistics Version 20.0 was used for all analyzes. The threshold for statistical significance for each test was set at 0.05.

## RESULTS

A total of 170 patients (132 AD and 38 control) were included in the study. The mean ages of AD patients and the control group were 70.83±9.35 (49-87) and 71.09±5.77 (63-84), respectively (p >0.05). 34.2% (n:13) of AD and 47.7% (n:63) of the control group were male (p >0.05). The mean MMSE was 28.04±1.16 (26-30) in the control group and 19.39±2.90 (10-23) in AD (p<0.05) (Table-I).

**Table-I:** Demographic and Laboratory data between AD and Control groups

	Control (n:38)	AD (n:132)	p
Age	71.09±5.77	70.83±9.35 (49-87)	0.43
Education (years)	5.67±5.42 (5-15)	7.12±6.10 (5-15)	0.25
MMSE	28.04±1.16 (25-30)	19.39±3.90 (10-24)	<0.001
Male/Female (n)	13/25	63/69	0.16
HT (n)	20	81	0.33
HL (n)	15	59	0.56
DM (n)	13	30	0.15
Fazekas (0/1/2/3) (n)	13/16/9/0	34/49/15/34	0.003
WBC	6.90±1.62 (4.4-10)	7.64±1.97 (3-13.1)	0.18
Neutrophil	56.63±7.06 (43.4-67.6)	62.24±7.64 (47.1-84.1)	0.013
Lymphocyte	32.02±7.14 (18.40-48.10)	25.59±7.20 (4.2-41.2)	0.004
NLR	1.9±0.66 (0.9-3.56)	2.96±2.46 (1.17-19.43)	0.005
Hemoglobin	17.59±24.2 (9.5-12.3)	13.06±1.82 (6.4-17)	0.35
Platelet	272.14±79.22 (133-474)	254.78±81.25 (67-475)	0.37
Vitamin D	15.64±9.62 (3.1-35.6)	15.49±8.97 (3.56-56.1)	0.93

*Abbreviations:* **MMSE:** Mini-mental state examination **AD:** Alzheimer’s Disease. **HT:** Hypertension. **HL:** Hyperlipidemia. **DM:** Diabetes Mellitus, **Fazekas:** Fazekas scoring, **WBC:** White blood cell, **NLR:** Neutrophil-Lymphocyte ratio.

Hypertension was found in 52.6% of the control (n:20) and 61.3% of the AD group (n:81) (p >0.05). Hyperlipidemia was detected in 39.4% of control (n:15) and 44.7% of AD patients (n:59) (p >0.05). Diabetes was presented in 34.2% of the control (n:13) and 22.7% of the AD group (n:30) (p >0.05) (Table-I).

The mean NLR was found 1.9±0.66 (0.9-3.56) in the control and 2.96±2.46 (1.17-19.43) in AD group (p<0.05) (Table-I). The receiver operating characteristic curve (ROC) analysis suggested that the optimum NLR cutoff point for AD was ≥1.91 65.2% sensitivity, 47.4% specificity) (Table-II). The mean value of Vitamin D was found 15.64±9.62 (3.1-35.6) ng/mL in the control and 15.49±8.97 (3.56-56.1) ng/mL in AD group (p>0.05) (Table-I).

**Table-II:** ROC Curve Analysis Between AD and Control Groups

Scale	AUC*	95% CI for AUC	Cut-point	Sensitivity	Specificity
NLR	0.649	0.548-0.750	≥1.91	65.2	47.4

*Abbreviations:* **AD:** Alzheimer’s Disease. **AUC:** Area under the ROC Curve. **NLR:** Neutrophil-Lymphocyte ratio.

In the second part of the study, AD subjects with mild ischemic lesions (Fazekas-1 and Fazekas-2) (n:64) and the control group were excluded. AD subjects without ischemic lesions (Fazekas-0) (n:34) and with severe ischemic lesions (Fazekas-3) (n:34) were compared again. Mean age was 66.22±8.55(52-76) years in Fazekas-0 and 72.12±12.13(49-86) years in Fazekas-3 group (p<0.05). 50% (n:17) of Fazekas-0 and 61.7% (n: 21) of Fazekas-3 were male (p>0.05) (Table-III). The mean MMSE was 20.63±3.45 (13-25) in Fazekas-0 and 16.81±4.33 (10-24) in Fazekas-3 group (p<0.05).

**Table-III:** Demographic and Laboratory data between Fazekas-0 and Fazekas-3 groups

	Fazekas-0 (n:34)	Fazekas-3 (n:34)	p
Age	66.22±8.55(52-76)	72.12±12.13(49-86)	0.001
Education (years)	7.43±6.11 (5-15)	5.91±6.47 (5-15)	0.16
MMSE	20.63±3.45(13-24)	16.81±4.33(10-24)	0.018
Male/Female (n)	17/17	21/13	0.32
HT (n)	12	26	0.001
HL (n)	14	23	0.028
DM (n)	6	8	0.54
WBC	8.00±1.94 (3.7-12.16)	7.61±1.45 (5.16-11.21)	0.29
Neutrophil	63.26±8.44 (50.2-84.1)	61.91±8.18 (35.8-81.6)	0.44
Lymphocyte	25.81±7.29 (6-35.4)	24.6±7.69 (4.2-42.6)	0.75
NLR	3.01±2.38 (1.57-13.63)	3.28±3.43 (0.84-19.43)	0.92
Hemoglobin	13.09±1.99 (6.4-16.1)	13.49±1.9 (9.2-17)	0.89
Platelet	279.90±95.74 (67-425)	268.25±52.76 (152-461)	0.61
Vitamin D	16.27±6.81 (4.6-29.7)	16.15±9.64 (4.7-35)	0.024

***Abbreviations:*** **Fazekas-0:** AD subjects without ischemic lesions, **Fazekas-3:** AD subjects with severe ischemic lesions, **MMSE:** Mini-mental state examination **HT:** Hypertension. **HL:** Hyperlipidemia. **DM:** Diabetes Mellitus, **WBC:** White blood cell, **NLR:** Neutrophil-Lymphocyte ratio

Hypertension was detected in 35.2% (n:12) of Fazekas-0 and 76.4% (n:26) of Fazekas-3 group (p <0.05). Hyperlipidemia was presented in 41.1% (n:14) of Fazekas-0 and 67.6% (n:23) of Fazekas-3 group (p <0.05). Diabetes mellitus was detected in 17.6% (n:6) of Fazekas-0 and 23.5% (n:8) of Fazekas-3 group (p >0.05) (Table-III).

The mean NLR was found 3.01±2.38 (1.57-13.63) in Fazekas-0 group and 3.28±3.43 (0.84-19.43) in Fazekas-3 group (p=0.92). The mean vitamin D was found 16.27±6.81(4.6-29.7) ng/mL in the Fazekas-0 and 16.15±9.64 (4.7-35) ng/mL in Fazekas-3 group (p=0.024) (Table-III). The ROC analysis showed that the optimum cutoff point concentration of vitamin D for Fazekas-0 was ≤11.65 (64.7% sensitivity. 54.1% specificity) (Table-IV).

**Table-IV:** ROC Curve Analysis Between Fazekas zero and Fazekas three Groups.

Scale	AUC*	95% CI for AUC	Cut-point	Sensitivity	Specificity
Vitamin D	0.627	0.524-0.729	≤11.65	64.7	54.1

***Abbreviations:* AD:** Alzheimer’s Disease.** AUC:** Area under the ROC Curve.

## DISCUSSION

In the literature, it was shown that both ischemia and inflammation play a role in the pathophysiology of AD.^6^ Vitamin D and NLR were used as a biomarker for inflammation and atherosclerosis in literature.^3^,^4^,^7^,^14^

In the first part of the present study showed that NLR was higher in AD than the control group, similar to previous studies.^3^,^18^ The optimum cut-off value of NLR for AD diagnosis was >1.91 (65.2% sensitivity, 47.4% specificity) in our study. Previous studies suggested that NLR can be a biomarker for AD diagnosis^3^,^18^ and, Kuyumcu et al. determined that the optimal cut-off value of NLR for AD diagnosis was >2.48 (69.29% sensitivity. 79.43% specificity).^3^ Dong et al. reported that the optimal cut-off value of NLR for AD diagnosis was >2.35 (83.0% sensitivity. 54.0% specificity).^18^ Both sensitivity and specificity of NLR were found lower in the current study than literature.^3^,^18^ NLR is not enough to identify AD, but it can be used as a helper biomarker similar to literature.

In second part of the present study, in the mean NLR there was no difference between Fazekas-0 and Fazekas-3 group (p=0.92). The previous study showed that NLR is associated with cerebral large-artery atherosclerosis but not with cerebral small-vessel disease.^7^ The current study detected that it wasn’t a link between NLR and WML similar to literature.

On the other hand, in the first part of the present study, vitamin D there was no difference between AD and control group. By contrast, a previous study showed that vitamin D level was lower in AD than control group^5^_._ In the second part of the current study, Fazekas-3 group had a lower concentration of vitamin D, a lower score of MMSE, more subjects with hypertension and hyperlipidemia than the Fazekas-0 group. The optimum cut-off value of Vitamin D for Fazekas-3 diagnosis was <11.65 (65.2% sensitivity, 47.4% specificity). According to results in first and second parts of the current investigation, vitamin D insufficiency was not connected to AD diagnosis but it was linked to a greater prevalence of ischemia. Fazekas-3 group had a lower score of MMSE, so ischemia may contribute to the worsening of AD symptoms.^19^ Hypovitaminosis-D has a greater risk of stroke in literature.^20^,^21^ Vitamin D may help to prevent neurodegenerative diseases of aging through protection against ischemia. Also, Fazekas-3 group had more subjects with hypertension in the present study. In literature, recommended therapeutic intervention for the treatment of hypertension is vitamin D replacement.^22^ As a result, vitamin D concentrations of Alzheimer’s patients should be closely monitored and supplementation should be given as soon as a deficiency is detected.

### Limitations:

The limitations of the study are listed as follows: Three Tesla MR which is more sensitive can be used instead of 1.5 Tesla MR. Cognitive examinations can be applied in more detail so a possible relationship between hypovitaminosis D and cognition can be found. Fazekas-3 group had higher rates of hypertension and hyperlipidemia in addition to hypovitaminosis-D. It can be conceivable to exclude patients who had a story about vascular risk factors like hyperlipidemia and hypertension for future studies.

## CONCLUSION

The present study showed NLR was higher in AD while there was no difference between the Fazekas-0 and Fazekas-3 AD groups. These data suggest that NLR is associated with AD but not ischemia. In clinical practice, NLR can be used as a biomarker for the diagnosis of AD. Vitamin D was no different between AD and the control group while it was lower in Fazekas-3 group than Fazekas-0 group. These data show that hypovitaminosis D may trigger ischemia in AD. In clinical practice, plasma vitamin D levels should be followed in patients with AD. If hypovitaminosis is detected, vitamin D has to replace for preventing ischemia.

### Authors’ Contribution:

**AE:** Conceived. designed. did statistical analysis, editing & writing of manuscript. Will be responsible, accountable for the accuracy and integrity of the work.

**ZSS & PBB:** Did data collection and manuscript writing.

**AE:** Did review and final approval of manuscript.
